# The decreased expression of TIPE2 protein in the decidua of patients with missed abortion and possible significance

**DOI:** 10.1186/s12958-017-0285-y

**Published:** 2017-08-29

**Authors:** Yingshuo Sun, Xiaoyan Wang, Yue Li, Han Sun, Lu Wan, Xishuang Wang, Lining Zhang, Zhenghui Fang, Zengtao Wei

**Affiliations:** 1 Department of Gynecology and Obstetrics, Clinical Medical School, Shandong University, 44# Wenhua Xi Road, Jinan, Shandong 250012 People’s Republic of China; 2 Department of Immunology, School of Basic Medical Sciences, Shandong University, 44# Wenhua Xi Road, Jinan, Shandong People’s Republic of China; 3 Department of Clinical Laboratory Services, Linyi People’s Hospital, Linyi, Shandong People’s Republic of China; 4 Department of Gynecology and Obstetrics, Jinan Central Hospital affiliated to Shandong University, 105# Jiefang Road, Jinan, Shandong 250013 People’s Republic of China

**Keywords:** Missed abortion, TIPE2, Cytokine, Hormone

## Abstract

**Background:**

Missed abortion is a common occurrence for otherwise healthy women. Immunological factor is one of the most important reasons. Tumor necrosis factor-α-induced protein-8 like-2 (TIPE2) is a novel negative immune regulator related to several human diseases. However, the expression level and clinical significance of TIPE2 in missed abortion remain unclear.

**Methods:**

The expression of *TIPE2* mRNA and protein in decidua and chorion from 36 missed abortion patients and 36 healthy controls was detected using quantitative real-time PCR, western blot and immunohistochemistry. In addition, serum TNF-ɑ and IL-10 levels were measured using flow cytometry. Serum estradiol and progesterone levels were measured by radioimmunoassay test. The correlations of TIPE2 protein levels with TNF-ɑ, IL-10, estradiol and progesterone were further analyzed.

**Results:**

TIPE2 protein levels were significantly lower in decidual tissues of missed abortion patients than those in healthy controls. The patients with missed abortion had significantly higher levels of serum TNF-ɑ, and lower levels of serum IL-10, estradiol and progesterone compared with healthy controls. The TIPE2 protein levels were positively related to serum IL-10 levels.

**Conclusion:**

Our data indicate TIPE2 could play important roles in maintaining the maternal-fetal tolerance and decreased TIPE2 expression in the decidua may be related to the development of missed abortion.

## Background

TIPE2, a member of the TNFAIP8 family, is a newly identified negative regulator of inflammation and immunity [1–4]. It is also called TNF-α-induced protein 8-like 2 (TNFAIP8L2) and highly expressed in murine inflamed tissues [1, 5]. It has been reported that mouse TIPE2 is preferentially expressed in hematopoietic cells, such as macrophages, B and T lymphocytes [1]. Its deficiency leads to multi-organ inflammation, splenomegaly and premature death in mice [1]. There are high levels of pro-inflammatory cytokines such as interleukin (IL)-1, IL-6, IL-12 and TNF-α as well as the inhibitory cytokine IL-10 in the serum of *TIPE2*-knockout mice [1]. However, human TIPE2 is expressed in both tissues of the immune system and non-immune tissues [6–10], including hepatocytes and neurons. Abnormal TIPE2 expression exists in patients with chronic inflammatory diseases and cancers, and correlates with the progression of diseases, which suggests that TIPE2 plays important roles in neoplastic and inflammatory diseases [11–15].

Missed abortion is defined as the arrest of embryonic or fetal development with ultrasound findings of an empty gestational sac or an embryo/fetus without cardiac activity [16]. It is a common occurrence for otherwise healthy women. At present, the multiple etiologic factors including genetic and uterine abnormalities, endocrine and immunological dysfunctions, infections, nutritional and environmental factors, psychogenetic factors, and endometriosis, have been identified [17–19]. Among them, an altered immunological environment associated with idiopathic early pregnancy demise should be emphasized [20]. It has been known that the genome of the embryo comes from the mother and the father equally, therefore, the fetus is a kind of semi- allograft for the mother [21, 22]. The regulation of immune system during pregnancy is essential for maintaining the immune tolerance to fetal semi-allograft and preventing the attack of immunologically distinct fetus by maternal immune system [23–26]. The association between missed abortion and inflammation or immunity is widely recognized, however, the expression status and role of human TIPE2 in patients with missed abortion remain unclear.

In the present study, we detected the expression status of TIPE2 in decidua and chorion of missed abortion patients and healthy controls at both mRNA and protein level by quantitative real-time PCR, western blot and immunohistochemistry. In addition, we also investigated Th1-type cytokine TNF-ɑ, Th2-type cytokine IL-10, estradiol and progesterone in the serum of patients with missed abortion and healthy controls, and evaluate the correlations of TIPE2 expression with cytokines, estradiol and progesterone in missed abortion patients. The results demonstrated that missed abortion patients have decreased expression of TIPE2 in decidual tissues. Altered expression of TIPE2 could contribute to the pathophysiology of missed abortion.

## Methods

### Human subjects

Thirty-six patients with missed abortion and 36 healthy controls with normal induced abortions were recruited from Department of Obstetrics and Gynecology in Jinan Central Hospital affiliated Shandong University from January to December, 2015. At first, we excluded pregnancies affected with significant chromosome abnormalities. All of the women examined had no any sign of autoimmune disorders, clinical genital infections or any other systemic disease, and hormone treatment in nearly three months. Gestation time (7–12 weeks) was evaluated based on the last menstrual period, and confirmed by ultrasound examination. All decidual and chorionic tissues were washed in 0.9% NaCl as soon as they had been removed from the uterus. Each sample was divided into two parts: one part was fixed overnight in 4% formaldehyde at room temperature for immunohistochemistry, and the other part was frozen in a − 80 °C refrigerator until protein and RNA extraction. The serum was collected before the participants were operated. The detailed demographic and clinical data were presented in Table 1. There are no statistically significant differences in the data, including maternal age, gestation age and gravity between missed abortion patients and healthy controls.

**Table 1:** The characteristics of missed abortion patients and healthy controls

Characteristics	Missed abortion patients	Healthy controls	*P* value
N	36	36	
Maternal age, years Gestational age, days Gravidity, orders	31.56±0.9546 48.35±1.492 2.094±0.217	29.00±1.522 47.11±1.80 1.926±0.337	>0.05 >0.05 >0.05

### RNA isolation, quantitative real-time PCR (qRT-PCR)

Total RNAs of decidua and chorion were extracted using a modified TRIzol one-step extraction method (TIANGEN, Beijing, China). The concentration of RNA was determined by ultraviolet absorption spectrometry. The same amount of RNA (2 μg) was reversely transcribed to cDNA using the Fast Quant RT Kit (TIANGEN, Beijing, China) according to the manufacture’s instruction. cDNA was used as template for the amplification of *TIPE2* gene. Real-time PCR was performed using UltraSYBR Mixture and *TIPE2* specific primers (CWBIO, Beijing, China) according to the manufacture’s protocol in the Bio-Rad CFX96. The sequences of *TIPE2* specific primers were as follows: forward 5′-GGAACATCCAAGGCAAGACTG-3′ and reverse 5′-AGCACCTCACTGCTTGTCTCATC-3′. The qRT-PCR reaction was performed according to the following conditions: pre-denaturation at 95 °C for 10 min, followed by 39 cycles of amplification at 95 °C for 15 s, 60 °C for 1 min, 65 °C for 5 s. Each sample was conducted in triplicate. Data were analyzed using the 2 − ∆∆Ct method. *TIPE2* data were normalized to the mRNA levels of housekeeping gene GAPDH. The primer sequences for GAPDH were shown as the following: forward 5′-AACGGATTTGGTCGTATTGGG-3′ and reverse 5′-CCTGGAAGATGGTGATGG GAT-3′.

### Western blot

Proteins were extracted from decidua and chorion using a modified TRIzol one-step extraction method. Protein concentration was detected by a BCA protein assay kit (Thermo Scientific, MA, USA). Total proteins were separated by 15% sodium dodecylsulfate–polyacrylamide gel electrophoresis and transferred to PVDF membranes (Millipore, Billerica, MA, USA). After blocking with 2% BSA in TBST containing 0.1% Tween-20 for 1 h at room temperature, the membrane was incubated overnight at 4 °C with 1:1000 dilution of anti-TIPE2 antibody (Abcam Cambridge, UK) and anti-β-actin antibody (ZSJQB Co., Ltd. Beijing, China), then was washed three times and incubated with 1:2000 dilution of secondary antibody (goat anti-rabbit IgG) for 1 h at room temperature. After washing, the membrane was visualized by ECL western blotting detection system (Amersham Biosciences, Little Chalfont, UK). β-actin was used as the loading control.

### Immunohistochemistry

The decidua and chorion were fixed in 4% formaldehyde and embedded in paraffin. Five μm-thick tissue sections were deparaffinized and rehydrated through decreasing concentrations (from 100% to 75%) of graded ethanol. Then, the specimens were treated with antigen microwave retrieval and endogenous peroxidase blocking and then blocked by 10% goat serum for 15 min at 37 °C. Rabbit monoclonal antibody for TIPE2 (1:60 dilution, Abcam, Cambridge, UK) was added to the sections separately overnight at 4 °C in a wet chamber. For negative controls, primary antibody was replaced by PBS. Then, the slides were incubated with a HRP-conjugated goat anti-rabbit IgG, followed dyaminobenzidine (DAB) staining using HistostainTM-Plus Kit (Gene Tech, Shanghai, China). Cytoplasmic staining of a brown-yellow granulation was considered indicative of positive results. Negative cells had clear cell structure without brown granulation in their cellular cytoplasm. High-resolution images of the stained sections were acquired using an Olympus DP72 digital camera and DP controller software (Olympus, Tokyo, Japan).

The staining intensity and positive expression area were evaluated in a blinded fashion by two independent pathologists. The staining intensity was divided into four grades: – (score 0), + (score 1), ++ (score 2), and +++ (score 3). The positive expression area was also classified into four categories: – (<1%, score 0), + (1–33%, score 1), ++ (34–66%, score 2), and +++ (67–100%, score 3). The sum of intensity and percentage scores was used as the final TIPE2 staining score. The expression of TIPE2 was defined as follows: no expression (total score 0); weak expression (total score 1 and 2); moderate expression (total score 3 and 4); strong expression (total score 5 and 6).

### Measurement of serum TNF-ɑ, IL-10, estradiol and progesterone levels

The blood samples were obtained before vacuum aspiration. The samples were put at 4 °C for blood clotting and the serum was acquired after centrifugation at 3000 rpm for 15 min at 4 °C. Then the serum was collected and frozen at −80 °C for TNF-ɑ, IL-10, estradiol and progesterone analysis. The levels of serum TNF-ɑ and IL-10 were measured with BD Cytometric Bead Array HUMAN Inflammation Kit (Becton, Dickinson and Company, USA) in accordance with the manufacturer’s instruction. Serum estradiol and progesterone levels were measured using Radioimmunoassay (RIA) (lodine (125 l)-Prog RIA Kit and lodine (125 l)-E2 RIA Kit) (JinDing, TianJin, China) on the Gamma Radioimmunoassay Counter.

### Statistical analysis

Data were presented as mean ± SEM. Differences between groups were analyzed by Unpaired Student’s t-test or chi-squared tests. Correlations were studied by Pearson’s correlation test. *p* < 0.05 was considered statistically significant. GraphPad Prism 5 software (La Jolla, CA, USA) was used to perform data analysis.

## Results

### The expression of TIPE2 mRNA in the decidua and chorion of missed abortion patients and healthy controls detected by real-time PCR

**Fig. 1:**
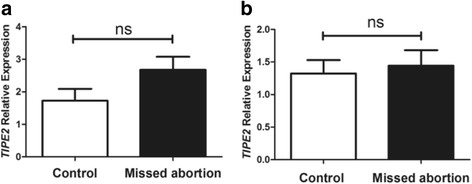
The expression of *TIPE2* mRNA in the decidua and chorion of 36 missed abortion patients and 36 healthy controls detected by quantitative real-time PCR. **a** The expression of *TIPE2* mRNA had no significant differences in decidual tissues between missed abortion patients and normal controls (*p* > 0.05); **b** No significant differences in *TIPE2* mRNA expression were observed in chorionic tissues between the two groups (*p* > 0.05)

Quantitative real-time PCR analysis was performed to detect the *TIPE2* mRNA expression in the decidua and chorion of missed abortion patients and healthy controls. However, no significant differences in *TIPE2* mRNA expression were observed in both decidua and chorion between the two groups (*p* > 0.05) (Fig. 1a and b).

### The expression of TIPE2 protein in the decidua and chorion of missed abortion patients and healthy controls detected by western blot

**Fig. 2:**
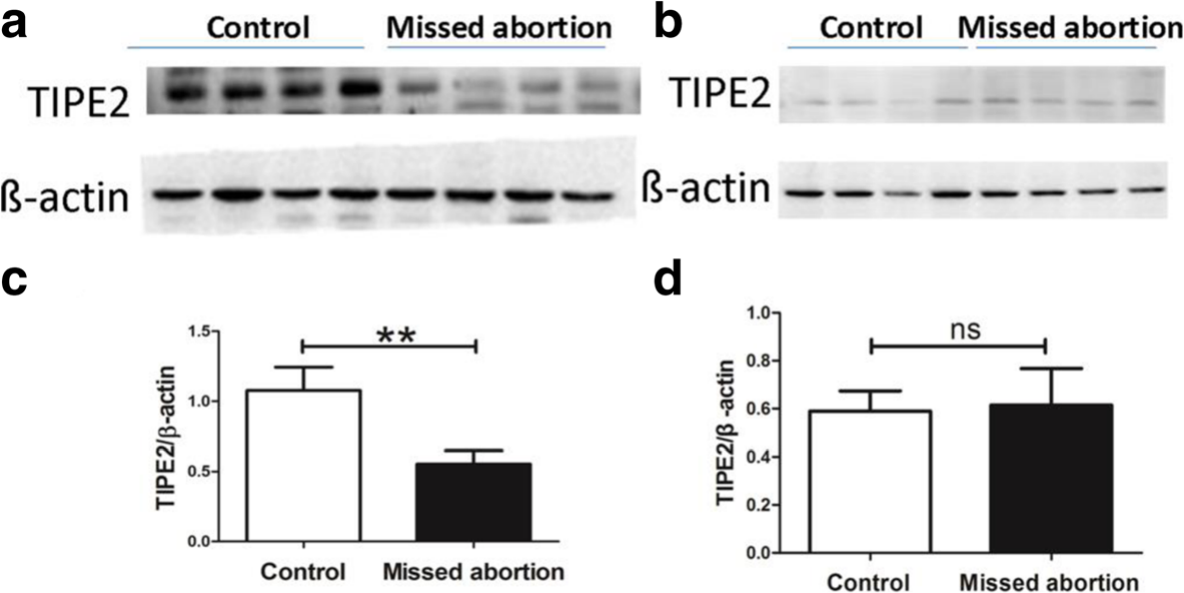
The expression of TIPE2 protein in the decidua and chorion of 36 missed abortion patients and 36 healthy controls detected by western blot. **a** The expression of TIPE2 protein in decidual tissues of missed abortion patients and healthy controls; **b** The expression of TIPE2 protein in chorionic tissues of missed abortion patients and healthy controls; **c** The expression of TIPE2 protein in the decidual tissues was significantly lower in missed abortion patients than that in healthy controls (*p* < 0.01); **d** No significant differences in the expression of TIPE2 protein were found in chorionic tissues between missed abortion patients and healthy controls (*p* > 0.05)

To investigate the expression status of TIPE2 proteins, we detected the protein levels of TIPE2 using western blot analysis in the decidua and chorion of missed abortion patients and healthy controls. The results revealed that the expression of TIPE2 protein in decidual tissues was significantly lower in missed abortion patients than that in healthy controls (*P* < 0.01) (Fig. 2a and c). However, no significant differences in the expression of TIPE2 protein were found in chorionic tissues between missed abortion patients and healthy controls (*P* > 0.05) (Fig. 2b and d). These data suggested the down-regulation of TIPE2 expression in decidual tissues of missed abortion patients.

### The expression of TIPE2 protein in the decidua and chorion of missed abortion patients and healthy controls detected by IHC

**Fig. 3:**
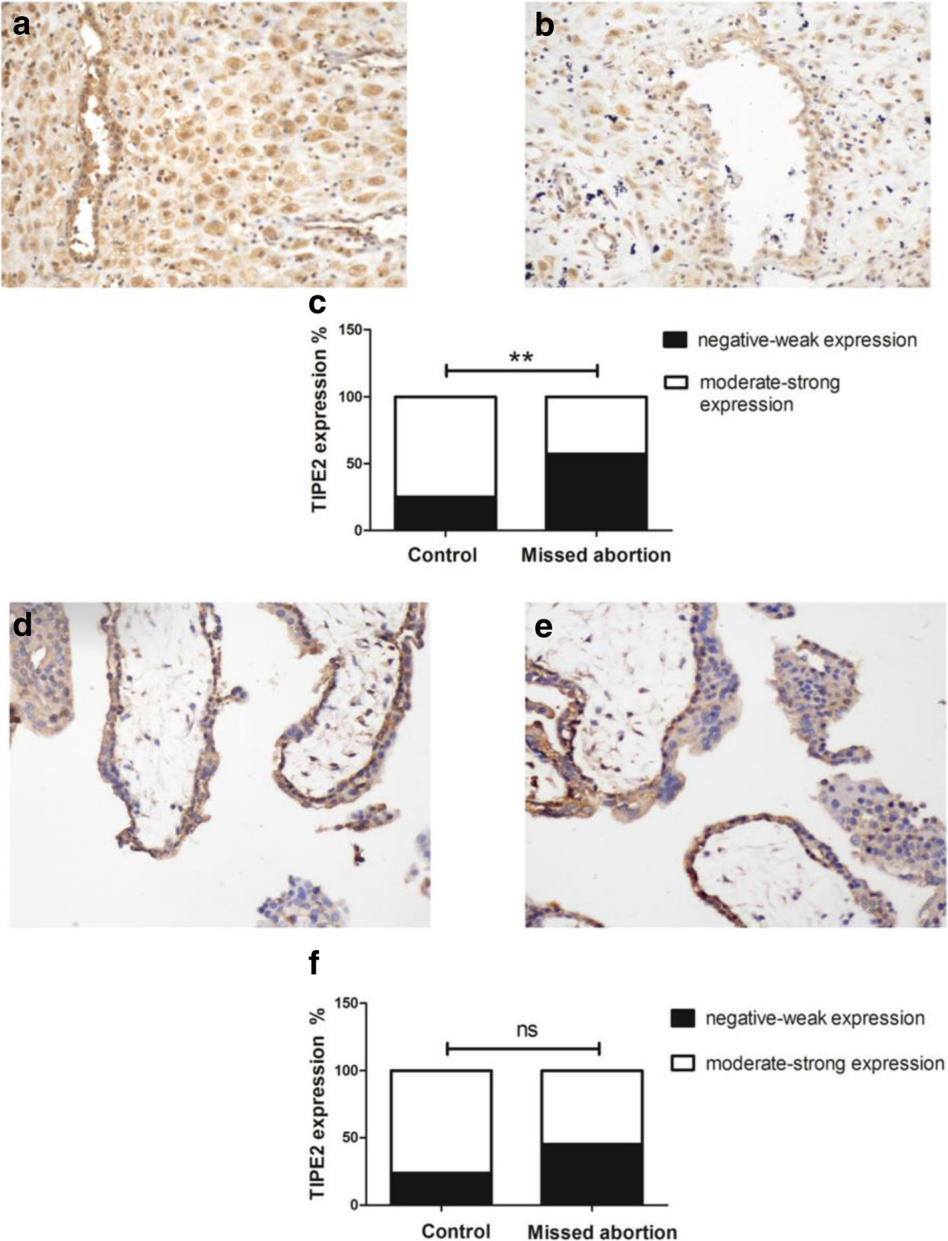
The expression of TIPE2 protein in the decidua and chorion of 36 missed abortion patients and 36 healthy controls detected by IHC. **a** The expression of TIPE2 protein in decidual tissues of missed abortion patients (original magnification, ×200); **b** The expression of TIPE2 protein in decidual tissues of healthy controls (original magnification, ×200); **c** TIPE2 positive staining was significantly lower in decidual tissues of missed abortion patients than that in healthy controls (*p* < 0.01); **d** The expression of TIPE2 protein in chorionic tissues of missed abortion patients (original magnification, ×200); **e** The expression of TIPE2 protein in chorionic tissues of healthy controls (original magnification, ×200); **f** No significant differences in TIPE2 expression were found in chorionic tissues between missed abortion patients and healthy controls (*p* > 0.05)

To further determine the expression sites and levels of TIPE2 proteins, we detected the TIPE2 proteins in the decidua and chorion of missed abortion patients and healthy controls by IHC. The results showed TIPE2 protein expressed in both decidua and chorion of missed abortion and control groups, TIPE2 positive staining localized in the cytoplasm of cytotrophoblasts and syncytiotrophoblasts, villous stromal cells, vessel endothelial cells of chorionic tissues, and decidual glandular epithelial cells, vessel endothelial cells and stromal cells of decidual tissues. By statistical analysis, we found that TIPE2 positive staining was significantly lower in decidual tissues of missed abortion patients than that in healthy control (*p* < 0.01; Fig. 3a-c). However, no significant differences in TIPE2 expression were found in chorionic tissues between missed abortion patients and healthy control (*P* > 0.05; Fig. 3d-f). These results further confirmed that TIPE2 expression was down-regulated in decidual tissues of missed abortion patients.

### Serum cytokine and hormone levels in missed abortion patients and healthy controls

**Fig. 4:**
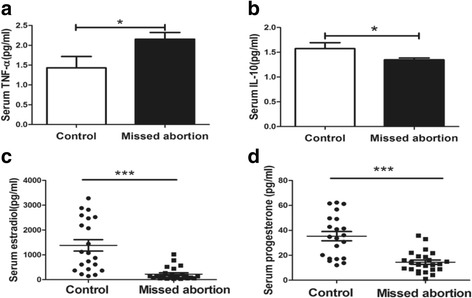
Serum cytokine and hormone levels in missed abortion patients and healthy controls. **a** Serum TNF-ɑ level in patients with missed abortion was obviously higher than that in healthy subjects (*p* < 0.05); **b** Serum IL-10 level was significantly lower in missed abortion patients than that in normal controls (*p* < 0.05); **c** Serum total estradiol level in missed abortion patients was significantly lower than that in healthy controls (*p* < 0.001); **d** Serum progesterone level was significantly decreased in missed abortion patients compared with healthy subjects (*p* < 0.001)

We next investigated the levels of Th1-type cytokine TNF-ɑ and Th2-type cytokine IL-10 in the serum of missed abortion patients and healthy subjects. The patients with missed abortion had significantly higher levels of serum TNF-ɑ (*p* < 0.05; Fig. 4a), and lower levels of serum IL-10 (*p* < 0.05; Fig. 4b) compared with healthy controls. In addition, serum estradiol and progesterone of gestation time (7–10 weeks) from the two groups were measured respectively by radioimmunoassay previously described. The results showed the levels of serum total estradiol (*p* < 0.001; Fig. 4c) and progesterone (*p* < 0.001; Fig. 4d) were significantly lower in missed abortion patients than those in healthy controls. These results indicated the important roles of increased Th1-type cytokines, reduced Th2-type cytokines, estradiol and progesterone in missed abortion.

### Correlation analysis of TIPE2 protein expression with serum TNF-ɑ, IL-10, estradiol and progesterone levels in missed abortion patients

**Fig. 5:**
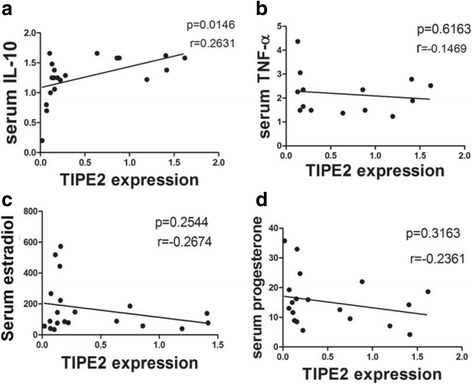
Correlation analysis of TIPE2 protein expression with serum TNF-ɑ, IL-10, estradiol and progesterone levels in missed abortion patients. **a** There was a significantly positive correlation between TIPE2 protein expression and serum IL-10 levels (*r* = 0.2631, *p* = 0.0146); **b** No statistically significant correlation was found between TIPE2 protein expression and serum TNF-ɑ levels (*r* = −0.1469, *p* = 0.6163); **c** The expression of TIPE2 had no significant relation to serum estradiol levels (*r* = −0.2674, *p* = 0.2544); **d** There was no statistically significant correlation between TIPE2 protein expression and serum progesterone levels (*r* = −0.2361, *p* = 0.3163)

To further determine the clinical significance of TIPE2 expression in missed abortion, we accordingly analyzed the correlations of TIPE2 protein expression with IL-10, TNF-ɑ, estradiol and progesterone. As shown in Fig. 5a, the expression level of TIPE2 protein was positively correlated with the serum level of IL-10 (*r* = 0.2631, *p* = 0.0146). However, we observed that the expression of TIPE2 had no significant relation to serum TNF-ɑ levels (*r* = −0.1469, *p* = 0.6163) (Fig. 5b). Furthermore, there were no statistically significant correlations between TIPE2 protein expression and serum estradiol (*r* = −0.2674, *p* = 0.2544) and progesterone levels (*r* = −0.2361, *p* = 0.3163) (Fig. 5c and d).

## Discussion

Missed abortion is one of the most common complications of pregnancy, several reasons have been identified for the failure of these pregnancies. Among them, immunologically mediated abortion has been paid much attention increasingly. It has been known that an immune suppressed state in maternal-fetal interface is necessary for the mother to tolerate the semi-allograft fetus, and the immunological mechanism occurring in maternal-fetal interface is very complex [27]. TIPE2, a newly identified immune inhibitor, manifests a negative regulatory effect in inflammation and the maintenance of immune homeostasis [1, 2, 8]. At present, abnormal expression of *TIPE2* mRNA and/or protein has been found in several chronic inflammatory diseases and cancers. On one hand, TIPE2 expression is down-regulated in peripheral blood mononuclear cells (PBMCs) of patients with SLE [11] and HBV [9] or HCV-induced hepatitis [12], as well as gastric cancer [13], hepatocellular carcinoma [14] and non-small cell lung cancer tissues [15]. On the other hand, it has been reported that TIPE2 expression is up-regulated in glomeruli from streptozotocin (STZ)-induced diabetic rats and renal biopsies of patients with diabetes [8], and PBMCs of chronic rejection patients [28], as well as renal cell carcinoma tissues [29], Non-Hodgkin’s Lymphoma [30]. Our previous study showed *TIPE2* mRNA and protein were both down-regulated in PBMCs of patients with childhood asthma [31]. These results suggest that TIPE2 may involve in the pathogenesis of some chronic inflammatory diseases and cancers, but the mechanism may be different. However, up to now, no reports have been made regarding the role of TIPE2 in missed abortion.

In the present study, we firstly collected the decidual and chorionic tissues of missed abortion patients and healthy controls, and detected the expression of *TIPE2* mRNA and protein in these tissues. *TIPE2* mRNA expression had no significant differences in the decidual and chorionic tissues between missed abortion patients and healthy controls. The results from western blot demonstrated that TIPE2 protein was down-regulated in the decidual tissues of missed abortion patients compared with healthy controls, while no significant difference in TIPE2 protein was observed in chorionic tissues between missed abortion patients and healthy controls. It has been reported that down-regulation of TIPE2 occurred at the protein but not at mRNA level, because RT-PCR revealed no significant difference in *TIPE2* mRNA between hepatocellular carcinoma and its adjacent tissues [25]. The reason for that may be the post-transcription modulation of TIPE2 expression.

Zhang L et al. reported that TIPE2 protein highly expressed in various types of cells, such as gland epithelial cells in colon and appendix, stratified squamous epithelium in esophagus and cervix [6]. To further investigate the expression sites and protein levels of TIPE2 in maternal-fetal interface, we measured the expression of TIPE2 in the decidual and chorionic tissues of missed abortion patients and healthy controls by IHC. We found that TIPE2 positive staining mainly exists in the cytoplasm of decidual glandular epithelial cells, vessel endothelial cells and stromal cells of decidual tissues. Cytotrophoblasts and syncytiotrophoblasts, villous stromal cells, vessel endothelial cells of chorionic tissues also express low levels of TIPE2 protein. The statistic results confirmed that TIPE2 protein was only reduced in the decidual tissues of missed abortion patients compared with healthy controls. The above data indicate TIPE2 could play important roles in maintaining the maternal-fetal tolerance and decreased TIPE2 expression in the decidua may be related to the development of missed abortion.

Cytokines form a complex regulatory network which maintains homeostasis between the fetus and maternal immune system. It has been reported that Th2-type cytokine IL-10 may play an important role in maternal tolerance of the fetus [32] and increases systemically in patients with a normal pregnancy compared with those with miscarriage. However, Th1-type cytokines, such as TNF-α and IFN-γ involved in triggering immunological pregnancy loss, i.e., death of embryos [33, 34]. The TNF-α level is markedly elevated in the serum of women with recurrent spontaneous miscarriage. Our results also showed higher serum TNF-ɑ and lower IL-10 levels in missed abortion patients compared with healthy controls. We further analyzed the correlations of TIPE2 with TNF-ɑ and IL-10, and found a positive correlation between TIPE2 protein and IL-10. However, no statistically significant correlation was observed between TIPE2 protein and TNF-ɑ. These results indicate increased Th1-type immune response in patients with missed abortion.

TIPE2 could inhibit inflammatory cells activation by down-regulating pro-inflammatory cytokines and cell mitosis, and the deletion of *TIPE2* in mouse results in fatal inflammation and high serum levels of pro-inflammatory cytokines such as interleukin (IL)-1, IL-6, IL-12 and TNF-α. However, the inhibitory cytokine IL-10 is also increased in the serum of *TIPE2*-knockout mice [1]. This is inconsistent with our result that there is a positive correlation between TIPE2 protein and IL-10. We speculate that the increased inhibitory cytokine IL-10 may be the results of feedback, while increased IL-10 could not enough to inhibit the effect of pro-inflammatory cytokines in *TIPE2*-knockout mice. Besides Th1/Th2 cells, the cytokines secreted by Th17/Treg cells also play important roles in recurrent pregnancy loss [35]. The correlations of TIPE2 with Th17/Treg cells needs to be further explored in the future.

M2 polarization is important for early successful pregnancies in humans [36]. It has been reported that TIPE2 could promote M2 macrophage differentiation through the activation of PI3K-AKT signaling pathway [37]. Therefore, TIPE2 could play important roles in supporting pregnancy by promoting M2 macrophage differentiation. In addition, trophoblast cell invasion into the maternal endometrium plays a crucial role during human embryo implantation and placentation [38]. However, some reports showed TIPE2 could inhibit the invasion of various cancer cells, including prostate cancer [39] and lung cancer cells [40]. Trophoblast cell invasion possesses limitation, and it is different from tumor cell invasion [41]. The exact effect of TIPE2 on trophoblast invasion remains to be clarified.

Hormonal levels in early pregnancy may have predictive value in regard to outcome of pregnancy. Aksoy S et al. reported that estradiol and progesterone levels in patients with missed abortion or anembryonic pregnancies were significantly lower than those in the normal group [42]. Moreover, it has been known that the major reproductive hormones, such as estradiol and progesterone, are critical modulators of immune reactions during pregnancy and in particular have a key role in inducing peripheral tolerance [43]. Here, we also found that the levels of estradiol and progesterone were significantly lower in missed abortion group compared with healthy controls. We further analyzed the associations of TIPE2 protein with estradiol and progesterone levels. However, there were no statistically significant correlations of TIPE2 protein expression with serum estradiol and progesterone levels.

## Conclusions

In conclusion, we demonstrated, for the first time, that TIPE2 protein was down-regulated in decidual tissues of patients with missed abortion, and positively related to the serum IL-10 levels. These data suggest that TIPE2 may be involved in maintaining the maternal-fetal tolerance. Understanding the exact mechanism will hopefully identify more new and effective strategies to diagnose and treat the patients with missed abortion.

## Abbreviations

IHC: Immunohistochemistry
IL: Interleukin
PBMC: Peripheral blood mononuclear cell
qRT-PCR: Quantitative real-time PCR
TIPE2: TNF-α-induced protein 8-like 2 (TNFAIP8L2)
TNF-α: Tumor necrosis factor-α
